# A signature of saliva-derived exosomal small RNAs as predicting biomarker for esophageal carcinoma: a multicenter prospective study

**DOI:** 10.1186/s12943-022-01499-8

**Published:** 2022-01-18

**Authors:** Kai Li, Yusheng Lin, Yichen Luo, Xiao Xiong, Lu Wang, Kameron Durante, Junkuo Li, Fuyou Zhou, Yi Guo, Shaobin Chen, Yuping Chen, Dianzheng Zhang, Sai-Ching Jim Yeung, Hao Zhang

**Affiliations:** 1 Institute of Precision Cancer Medicine and Pathology, School of Medicine, and Department of General Surgery, The First Affiliated Hospital of Jinan University, Jinan University, Guangzhou, Guangdong China; 2 Institute of Precision Cancer Medicine and Pathology, Jinan University Medical College, Guangzhou, Guangdong China; 3 Department of Hematology, University Medical Center Groningen, University of Groningen, Groningen, the Netherlands; 4 Graduate School, Shantou University Medical College, Shantou, Guangdong China; 5 Department of Bio-Medical Sciences, Philadelphia College of Osteopathic Medicine, 4170 City Ave, Philadelphia, PA 19131 USA; 6 Department of Thoracic Surgery, Anyang Tumor Hospital, Anyang, Henan China; 7 Endoscopy Center, Affiliated Cancer Hospital of Shantou University Medical College, Shantou, Guangdong China; 8 Department of Thoracic Surgery, Affiliated Cancer Hospital of Shantou University Medical College, Shantou, Guangdong China; 9 Department of Emergency Medicine, University of Texas MD Anderson Cancer Center, Houston, TX USA; 10 Department of General Surgery, The First Affiliated Hospital of Jinan University, and Institute of Precision Cancer Medicine and Pathology, School of Medicine, Jinan University Medical College, 601 Huangpu Avenue West, Guangzhou, 510632 Guangdong China; 11 Minister of Education Key Laboratory of Tumor Molecular Biology, Jinan University, Guangzhou, 510632 China

**Keywords:** Transfer RNA-derived small RNA, tRNA-derived fragments, Sequencing of salivary extracellular vesicles, Liquid-biopsy signature, Pre-operative biomarker of adjuvant therapy, Esophageal carcinoma

## Abstract

**Background:**

The tRNA-derived small RNAs (tsRNAs) are produced in a nuclease-dependent manner in responses to variety of stresses that are common in cancers. We focus on a cancer-enriched tsRNA signature to develop a salivary exosome-based non-invasive biomarker for human esophageal squamous cell carcinoma (ESCC).

**Methods:**

Cancer-enriched small RNAs were identified by RNA sequencing of salivary exosomes obtained from ESCC patients (*n* = 3) and healthy controls (*n* = 3) in a pilot study and further validated in discovery cohort (*n* = 66). A multicenter prospective observational study was conducted in two ESCC high-incidence regions (*n* = 320 and 200, respectively) using the newly developed biomarker signature.

**Results:**

The tsRNA (tRNA-GlyGCC-5) and a previously undocumented small RNA were specifically enriched in salivary exosomes of ESCC patients, ESCC tissues and ESCC cells. The bi-signature composed of these small RNAs was able to discriminate ESCC patients from the controls with high sensitivity (90.50%) and specificity (94.20%). Based on the bi-signature Risk Score for Prognosis (RSP), patients with high-RSP have both shorter overall survival (OS) (HR 4.95, 95%CI 2.90–8.46) and progression-free survival (PFS) (HR 3.69, 95%CI 2.24–6.10) than those with low-RSP. In addition, adjuvant therapy improved OS (HR 0.47, 95%CI 0.29–0.77) and PFS (HR 0.36, 95%CI 0.21–0.62) only for patients with high but not low RSP. These findings are consistent in both training and validation cohort.

**Conclusions:**

The tsRNA-based signature not only has the potential for diagnosis and prognosis but also may serve as a pre-operative biomarker to select patients who would benefit from adjuvant therapy.

**Trial registration:**

A prospective study of diagnosis biomarkers of esophageal squamous cell carcinoma, ChiCTR2000031507. Registered 3 April 2016 - Retrospectively registered.

**Supplementary Information:**

The online version contains supplementary material available at 10.1186/s12943-022-01499-8.

## Background

Esophageal squamous cell carcinoma (ESCC) is ranked seventh for cancer morbidity and sixth for cancer mortality worldwide [1]. Patients often present at an advantage stage with lymph node metastasis at the time of diagnosis, which leads to a 5-year survival rate of approximately 20% [2–4]. To maximize the chance for eligibility for curative surgical resection, early detection and diagnosis of ESCC is expected to be important. Currently, biomarkers suitable for detection of early stage ESCC are lacking. Besides, loco-regional recurrence occurs in 30 to 40% of patients after surgical resection with intention to cure. Adjuvant radiotherapy and chemotherapy was important for ESCC, but their clinical benefit is controversial [5–7]. There are no biomarkers for predicting benefits of adjuvant therapies for ESCC either. Thus, early detection of patients and more precise stratification to guide adjuvant treatments are urgently needed for this malignancy.

Endoscopic examination with biopsy is invasive, and imaging studies are insensitive as screening modalities for ESCC. Minimally invasive technologies such as cytosponge or transnasal endoscopy have cost and discomfort barriers to wide-spread acceptance as screening methods for ESCC. Recently, liquid biopsy has been widely investigated for non-invasive cancer detection, and it is mainly based on three core types of biological materials originating from the cancer: circulating tumor cells (CTCs), circulating tumor DNA (ctDNA), and exosomes [8]. Despite their potential, the use of ctDNA and CTC as liquid biopsy methods have several limitations. Given that the fraction of ctDNA in total cell-free DNA is usually scarce, often < 0.01% [9, 10], detection sensitivity is a serious concern, especially for early cancer detection [10]. In addition, the translation of CTC into clinical practice is limited by challenges of their isolation due to extreme rarity, fragility, and oncogenetic/phenotypic heterogeneity [10, 11]. In contrast, exosomes are a type of extracellular vesicles containing proteins, DNAs, and RNAs representative of many characteristics of the cells from which they are secreted [12]. Exosomes are secreted by various types of cells and reflect heterogeneous biological changes associated with the tumors.

Exosomes contain many types of small RNA, such as miRNA, piwi-interacting RNA (piRNA), small nucleolar RNA (snoRNA), tRNA-derived small RNAs (tsRNAs), and other unidentified small RNAs [13–16]. Although miRNAs are the most studied category of small RNA biomarkers in exosomes, other exosome-derived small RNAs are emerging as novel cancer-enriched diagnostic and prognostic biomarkers [16, 17]. tsRNAs (also called tRNA-derived fragments (tRFs)), which were consider to be degradation products initially, are novel small non-coding RNAs (sncRNAs) generated from precursor or mature tRNAs [18–20]. tsRNAs were produced in a nuclease (angiogenin, RNY1, Dicer) dependent manner in response to stress such as amino acid starvation, oxidative stress and hypoxia [18, 21, 22]. It’s easy to conflate tsRNAs with cancer since the tumor microenvironment is characterized by hypoxia and nutrient deficiency. Recent studies have found that tsRNAs are dysregulated in various types of cancer [23–25]. However, the exploration of the potentials of tsRNA-based liquid biopsy is at an early stage [23–25].

Compared to ctDNA and CTCs that require phlebotomy to obtain liquid biopsy samples, exosomes are present in virtually all bodily fluids such as blood, saliva, urine, and cerebrospinal fluid, broadening the choices of sample sources for liquid biopsy. We previously developed salivary exosome-based detection of chimeric RNAs and mRNAs as a non-invasive liquid biopsy method for diagnosis and monitoring the progression of diseases [26–28].

In this study, by comparing the small RNAs in salivary exosomes of ESCC patients with that of healthy controls, we discovered two cancer-enriched small RNAs, tRNA-GlyGCC-5 and a previously uncharacterized small RNA for which we coined the name “small RNA identified in Exosome from Saliva of ESCC patients” (sRESE). The bi-signature composed of the levels of these two salivary exosomal small ncRNAs (abbreviated as sesncRNAs) were evaluated for their potential as an ESCC biomarker in a prospective multicenter observational study.

## Methods

### Study population

**Fig. 1:**
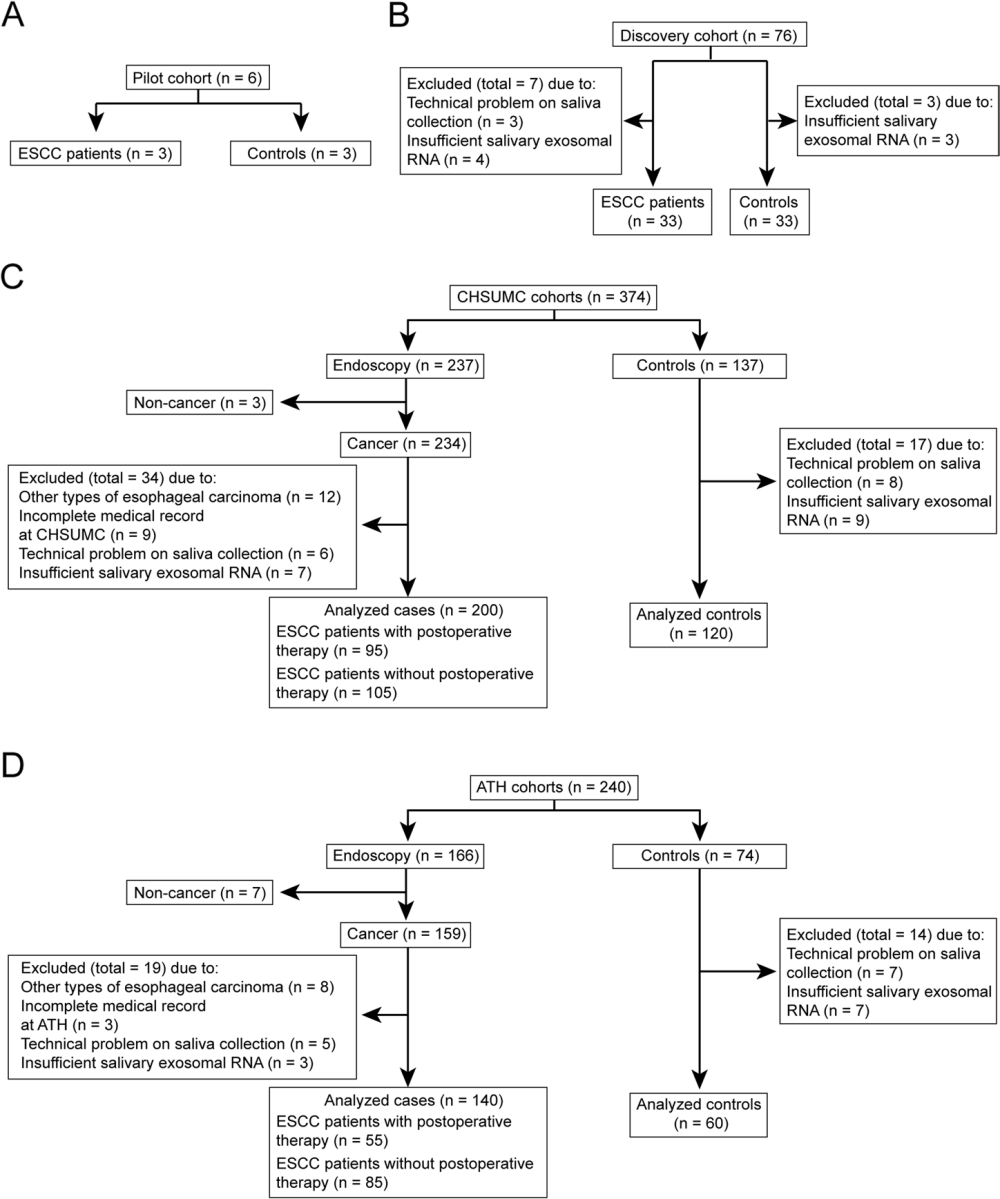
Flow diagrams showing the design of the pilot cohort, discovery cohort and the two patient cohorts. **A** The pilot cohort. **B** The discovery cohort. **C** The CHSUMC training cohort. **D** The ATH validation cohort

The study includes a pilot cohort (3 ESCC patients and 3 controls) and a discovery cohort (33 ESCC patients and 33 controls) as detailed in Fig. 1A and B. A prospective multi-cohort clinical study (ChiCTR2000031507) was registered on the Chinese Clinical Trial Registry (http://www.chictr.org.cn). This study involves prospective observational cohorts from two institutions: The Cancer Hospital of Shantou University Medical College (CHSUMC, Shantou, Guangdong, China) and Anyang Tumor Hospital (ATH, Anyang, Henan, China). As of January 1, 2018, we recruited 237 patients who was scheduled to undergo endoscopy and 137 healthy volunteers at CHSUMC, 166 patients who was scheduled to undergo endoscopy and 74 healthy volunteers at ATH. A total of 614 saliva samples were collected. The CHSUMC cohort was for constructing the diagnostic and prognostic models, and the ATH cohort was for model validation. Inclusion and exclusion criteria of those two cohorts are shown in Fig. 1.

These studies were conducted under clinical protocols approved by the Institutional Ethics Committees and Review Board of Cancer Hospital of Shantou University Medical College (CHSUMC) (IRB serial number: #04–070) and Anyang Tumor Hospital (ATH) (AZLL022016008161201). We obtained written informed consents from all participants per the principles established by the Helsinki Declaration. The cases recruited in this study were all newly diagnosed ESCC without prior anticancer treatment. The median follow-up time was 37 months (range: 2–63). All healthy subjects were approached for participation in the study in public spaces (e.g., parks, senior activity centers, and shopping areas) of the respective cities and matched to at least one ESCC case for gender, age, and tobaccos use. The healthy controls were excluded if they had any history of malignancy, severe oral disease, diabetes mellitus, lung disease, renal or hepatic dysfunction, severe immune alterations, and cardiovascular event in the past 6 months.

### Clinicopathological characteristics and clinical outcomes

The demographic and clinical data (age, sex, pathologic diagnosis, cancer treatment, etc.) were obtained from electronic medical record databases. The pathologic staging was done according to the Union for International Cancer Control (UICC) Tumor-Node-Metastasis (TNM) staging system (7th edition) [29]. Stage I and II were classified as the early-stage, and stage III and IV the late-stage. Progression-free survival (PFS) was defined as the duration from the time of ESCC primary surgery to the first relapse at any body site or death of any causes, whichever occurred first. Overall survival (OS) was defined as the duration from ESCC diagnosis to death of any causes.

### Statistical analysis

Comparisons between independent groups were performed with the t test or one-way ANOVA with post hoc intergroup comparisons, where appropriate [30, 31]. Data were tested for normal distribution using the Shapiro-Wilk test, and the Brown-Forsythe test was used to evaluate for equal variance. For non-parametric comparison between two groups, a rank-sum test (Wilcoxon matched-pairs signed-ranks test) would be used.

The sample size of the discovery cohort was determined a priori. Based on an estimated accrual interval of 36 months and additional follow-up after the accrual interval of up to 60 months, 33 cases and 33 controls would be needed to reject the null hypothesis. Their survival curves were equal with a power > 0.800 if the median survival of patients was 40 months and that the controls were > 60 months. The Type I error probability for the analysis was 0.05.

The differences of proportions in clinicopathological characteristics were analyzed with the Chi-square test, and the correlations between continuous variables were evaluated using Pearson’s correlation test. The area under receiver operating characteristics curve (AUROC) was used to assess the predictive performance of sesncRNAs. The optimal cutoff value for classification using sesncRNAs was based on the Youden index.

Survival analyses used the Kaplan-Meier method and were compared by the log-rank test as well as univariate and multivariate Cox proportional hazards modeling. A backward stepwise approach was applied in the discovery phase to identify the highly predictive sesncRNAs. Final Cox proportional hazards models were constructed using a sesncRNA-signature. Age, gender, histologic differentiation, tumor length, and stage as covariates, and the models were evaluated for validity by the Score test and calculating Martingale and Schoenfeld residuals using R package “ggcoxdiagnostics.” A nomogram was formulated based on the results of multivariate analysis using the R package “rms.” The performance of the nomogram was assessed by the concordance index (C-index) and by comparing nomogram-predicted survival with Kaplan-Meier estimates of survival probability [32].

We used G*power (https://www.psychologie.hhu.de/ arbeitsgruppen/allgemeine-psychologie-und-arbeitspsychologie/gpower.html) for a priori estimation of sample size [33]. All other statistical analyses were conducted using R, version 3.5.2 (R Foundation for Statistical Computing, Vienna, Austria. http://www.R-project.org/). A *P* value of < 0.05 was considered to be significant, and all tests were 2-sided.

Details for sample collection and experimental process are included in supplementary materials and methods.

## Results

### Discovery

**Fig. 2:**
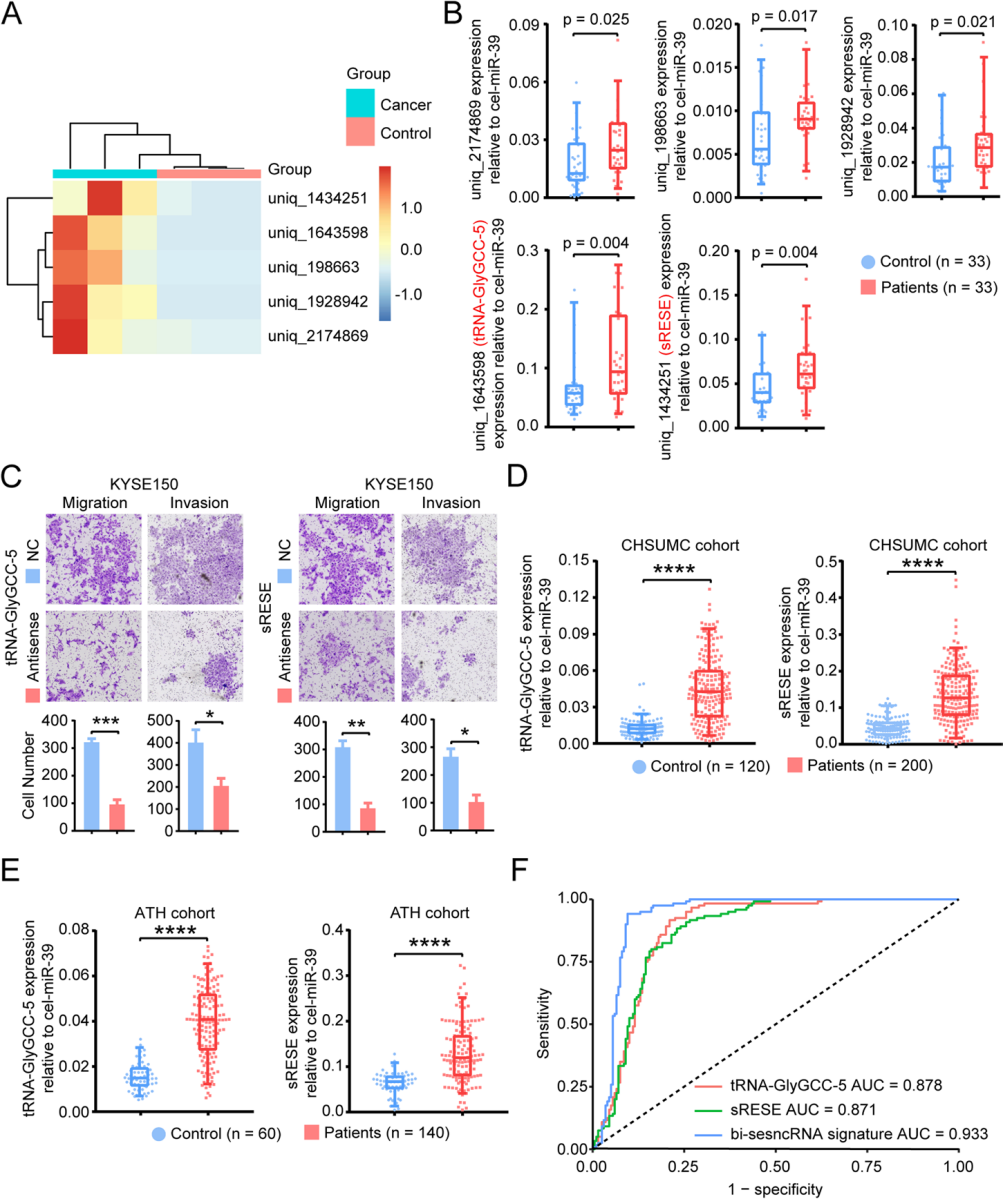
Identification of cancer-enriched sesncRNAs in salivary exosomes of ESCC patients. **A** The heatmap showing the top five differently expressed small RNAs by small RNA-seq of salivary exosomes. **B** Confirmation of the differentially expressed salivary RNAs. **C** Effect of sesncRNAs on cell migration and invasion. All experiments were performed in biological triplicate. **D**-**E** The box and scatter plots of tRNA-GlyGCC-5 (left) and sRESE (right) in the CHSUMC cohort (**D**) and the ATH cohort (**E**). **F** The results of ROC analysis of sesncRNAs in the CHSUMC cohort. SEM (*p < 0.05, **p < 0.01, ***p < 0.001, ****p < 0.0001 by unpaired t-test)

For the pilot study of 3 ESCC patients and 3 healthy volunteers (Fig. 1A), the isolated exosomes from either saliva or cell lysate were confirmed by TEM (Fig. S1A) and immunoblot using antibodies against specific exosomal markers (Alix, TSG101, CD63, CD9) and Calnexin, an intracellular protein that is not present in exosomes (Fig. S1B). Nanoparticle tracking showed that the average diameter of exosomes from saliva was 95 nm (Fig. S1C). Compared to the controls, 1366 differentially (*p* < 0.05) expressed sesncRNAs (excluding miRNAs) were identified in ESCC patients. Among them, 32 were highly expressed in ESCC patients with log_2_ fold changes > 2, and the top five candidates with the most significant fold changes were selected for further investigation (Fig. 2A).

Totally, 33 ESCC patients and 33 controls were recruited at CHSUMC to evaluate the levels of the top five sesncRNAs by RT-qPCR (Fig. 1B). Two of the five sesncRNAs met the predetermined significance level of 0.01 (Fig. 2B). Both of them, tsRNA (tRNA-GlyGCC-5) [34] and a previously uncharacterized small non-coding RNA, located in chromosome 1. Blast analysis on the National Center for Biotechnology Information (NCBI) found that tRNA-GlyGCC-5 is derived from 5’end of TRG-GCC. Further analysis found that sRESE gene resided between *SSX2IP* and lncRNA *LOC102724892.* Since this uncharacterized RNA is a small RNA identified in Exosomes from the Saliva of ESCC patients, we named it sRESE. The expression levels of these two sesncRNAs were examined in exosomes (Fig. S2A) and cell lines (Fig. S2B). Compared to the immortalized esophageal epithelial cells, both tRNA-GlyGCC-5 and sRESE were highly expressed in exosomes secreted into the conditioned media and ESCC cell lines. Sanger sequencing furthermore confirmed that these sesncRNAs were present and detectable in exosomes (Fig. S3). To understand the biological role of these sesncRNAs, we transfected ESCC cells with antisense RNAs against sesncRNAs, and found that proliferation, migration, and invasion were all significantly suppressed in both KYSE150 (Fig. 2C) and TE-12 cells (Fig. S4) suggesting that these sesncRNAs could be involved in the regulation of cell proliferation, migration, and invasion.

### Detection of the presence of ESCC

A prospective study (ChiCTR2000031507) is currently underway to collect saliva samples to study exosomal biomarkers. Since the pre-specified sample size has not been reached, the study is still ongoing. The demographic and clinicopathological characteristics of the patients in this interim analysis were summarized in Table 1. We analyzed the levels of these sesncRNAs in the salivary samples collected as of January 1, 2018 (Fig. 1C and D) and found that both tRNA-GlyGCC-5 and sRESE were significantly (*p* < 0.001) increased in ESCC patients compared with healthy volunteers in both CHSUMC cohort (200 ESCC patients and 120 controls, Fig. 2D) and ATH cohort (140 ESCC patients and 60 controls, Fig. 2E). In addition, the AUROC for tRNA-GlyGCC-5 and sRESE is 0.878 and 0.871, respectively, in the CHSUMC subjects (training cohort) (Fig. 2F). Using the optimal cutoff values for tRNA-GlyGCC-5 and sRESE determined using the Youden indices in the receiver operating characteristics analyses of the training cohort, the sensitivity of tRNA-GlyGCC-5 and sRESE in the prediction of ESCC is 79.00 and 77.00%, respectively, for the training cohort (Table 2).

**Table 1:** Patient demographics and clinicopathological characteristics of the training and validation cohorts

	CHSUMC cohort		*P* value	ATH cohort		*P* value	*P* value
Variables	Healthy	Patient		Healthy	Patient		
	(*n* = 120)	(*n* = 200)		(*n* = 60)	(*n* = 140)		
	*n* (%)	*n* (%)		*n* (%)	*n* (%)		
Age (years)	60.69 ± 8.66	61.06 ± 9.05	0.720^B^	61.13 ± 9.20	61.24 ± 8.65	0.936^B^	0.852^B^
Gender							
Female	50 (41.7)	74 (37.0)	0.410^A^	25 (41.7)	47 (33.6)	0.335^A^	0.566^A^
Male	70 (58.3)	126 (63.0)		35 (58.3)	93 (66.4)		
Tumor depth^C^							
T1/T2	NA	58 (29.0)		NA	52 (37.1)		0.126^A^
T3/T4	NA	142 (71.0)		NA	88 (62.9)		
Lymph node metastasis							
Negative	NA	85 (42.5)		NA	70 (50.0)		0.185^A^
Positive	NA	115 (57.5)		NA	70 (50.0)		
Histological differentiation							
Poor	NA	41 (20.5)		NA	18 (12.9)		0.179^A^
Moderate	NA	99 (49.5)		NA	78 (55.7)		
Well	NA	60 (30.0)		NA	44 (31.4)		
Largest tumor dimension (cm)	NA	4.86 ± 1.45		NA	5.01 ± 1.44		0.319^B^
TNM Stage							
I/II	NA	78 (39.0)		NA	66 (47.1)		0.148^A^
III/IV	NA	122 (61.0)		NA	74 (52.9)		

For categorical variables, the number of patients was shown as n (%); quantitive variables were mean ± SD

^A^χ2 test was used for comparing control and patient group

^B^Unpaired t test was used for comparing control and patient group

^C^Tumor depth indicated that how deeply tumor cells have invaded

**Table 2:** Performance of sesncRNAs test to differentiate ESCC patients from healthy subjects in CHSUMC and ATH cohorts

Variables	Cohorts	Cancer	Test Positive (*n*)	Test Negative (*n*)	Total (*n*)	Sensitivity	Specificity	PPV	NPV
tRNA-GlyGCC-5	CHSUMC	Absent	10	110	120	79.00%	91.67%	94.05%	72.37%
		Present	158	42	200				
		Total	168	152	320				
sRESE	CHSUMC	Absent	14	106	120	77.00%	88.33%	91.67%	69.73%
		Present	154	46	200				
		Total	168	152	320				
Bi-sesncRNA signature	CHSUMC	Absent	7	113	120	90.50%	94.20%	96.28%	85.61%
		Present	181	19	200				
		Total	188	132	320				
	ATH	Absent	9	51	60	87.14%	85.00%	93.13%	75.00%
		Present	122	18	140				
		Total	131	68	200				

*PPV* positive predictive value, *NPV* negative predictive value

The cutoff value calculated in CHSUMC cohort was applied in the ATH cohort

Test Positive in this analysis is based on a sesncRNA level or RSD higher than cutoff value; the remaining individuals were classified as Test Negative

To investigate the efficacy of a bi-sesncRNA signature (tRNA-GlyGCC-5 and sRESE), we performed a logistic regression analysis using the expression values of tRNA-GlyGCC-5 and sRESE to predict the presence of ESCC. The bi-sesncRNA signature risk score for diagnosis (RSD) was defined as:

$$\mathrm{RSD}=111.01\ \mathrm{x}\ \left(\mathrm{expression}\ \mathrm{value}\ \mathrm{of}\ \mathrm{tRNA}-\mathrm{GlyGCC}-5\right)+27.198\ \mathrm{x}\ \left(\mathrm{expression}\ \mathrm{value}\ \mathrm{of}\ \mathrm{sRESE}\right)-4.029.$$

ROC analysis indicated that the bi-sesncRNA signature RSD has better performance than each sesncRNA alone (AUROC 0.933 vs. 0.878 or 0.871, Delong test, both *p* < 0.001). Based on the optimal cutoff value (0.040) of the Youden index obtained from the ROC curve, ESCC patients in the CHSUMC cohort could be discriminated from controls by the RSD with a sensitivity of 90.50% and a specificity of 94.20%. Additionally, the positive predictive value (PPV) was 96.28%, and the negative predictive value (NPV) was 85.61% (Table 2). The cutoff value from the CHSUMC training cohort was then applied in the ATH validation cohort and found that the sensitivity, specificity, PPV, NPV are 87.14, 85.00, 93.13, 75.00%, respectively (Table 2). Interestingly, based on this cutoff, patients with stage I ESCC could also be discriminated from controls both in CHSUMC and ATH cohorts (Table S1). Therefore, the bi-sesncRNA signature could robustly distinguish ESCC (including stage I disease) patients from healthy subjects, thus promising a high translational potential.

### Prognostic prediction of ESCC

To assess the potential clinical utility of a bi-sesncRNA signature score in ESCC prognosis, we developed logistic regression formula to model the prediction of vital status to calculate a Risk Score for Prognosis (RSP) for each patient based on the two sesncRNAs expression levels:

$$\mathrm{RSP}=22.979\ \mathrm{x}\ \left(\mathrm{expression}\ \mathrm{value}\ \mathrm{of}\ \mathrm{tRNA}-\mathrm{GlyGCC}-5\right)+5.741\ \mathrm{x}\ \left(\mathrm{expression}\ \mathrm{value}\ \mathrm{of}\ \mathrm{sRESE}\right)-2.199.$$

**Fig. 3:**
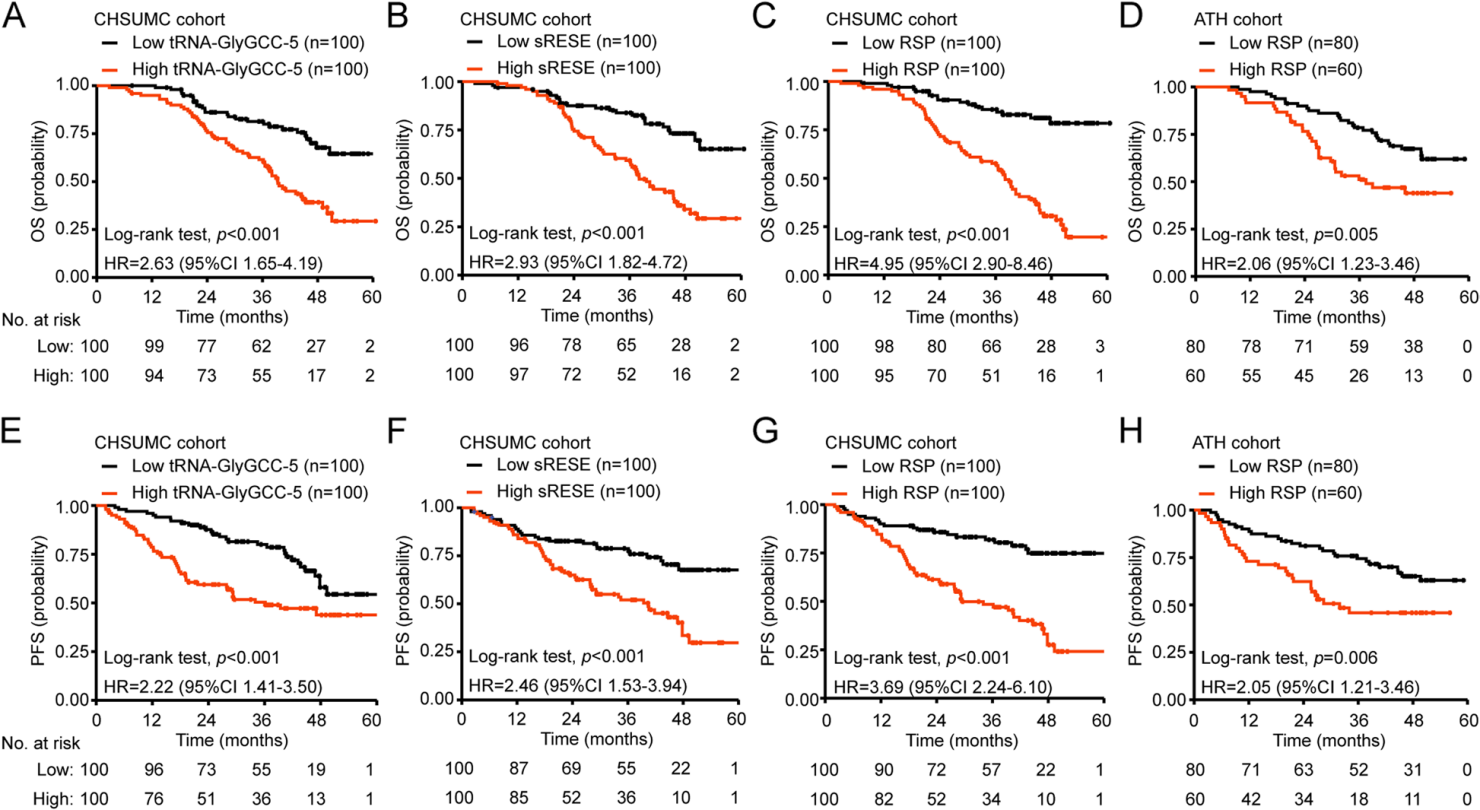
Performance of sesncRNAs for prognostication in CHSUMC and ATH cohorts. **A-B** and **E-F** Kaplan-Meier analysis shows that the OS and PFS were significantly longer in patients with low expression of tRNA-GlyGCC-5 (**A** and **E**) or sRESE (**B** and **F**) than those with high expression. **C** and **G** The sesncRNAs-based Risk Score for Prognosis (RSP) of each patient. Kaplan-Meier analysis shows that patients with low-RSP have longer OS (**C**) and PFS (**G**) than those with high-RSP in the CHSUMC cohort. **D** and **H** OS (**D**) and PFS (**H**) were significantly longer in patients with low RSP than those with high RSP in the ATH cohort. The *p*-values were calculated using the unadjusted log-rank test and hazard ratios (HR) using univariate Cox regression. CI, confidence interval

The medians of tRNA-GlyGCC-5, sRESE, and bi-sesncRNA signature RSP were used to divide the patients in the CHSUMC cohort into high (above median) and low (at or below median) groups. The bi-sesncRNA signature RSP is highly correlated with the lymph node metastasis, and histological differentiation (Table 3). Kaplan-Meier analysis revealed that ESCC patients with high-RSP have a significantly shorter OS (Fig. 3C, HR = 4.95, 95%CI 2.90–8.46, *p* < 0.001) and PFS (Fig. 3G, HR = 3.69, 95%CI 2.24–6.10, *p* < 0.001) than those with a low-RSP. Notably, the bi-sesncRNA signature improved the prediction of OS and PFS than either sesncRNA alone (Fig. 3A and B, OS: HR 4.95 [95%CI 2.90–8.46] vs 2.63 [1.65–4.19] or 2.93 [1.82–4.72]; PFS: HR 3.69 [2.24–6.10] vs 2.22 [1.41–3.50] or 2.46 [1.53–3.94]).

**Table 3:** Association of tRNA-GlyGCC-5 expression, sRESE expression and bi-sesncRNAs signature RSP with demographic and clinicopathological characteristics of the CHSUMC cohort

Variables	Patients: *n*	tRNA-GlyGCC-5 level		*P* value	sRESE level		*P* value	Bi-sesncRNA signature		*P* value
		Low: *n* (%)	High: *n* (%)		Low: *n* (%)	High: *n* (%)		Low: *n* (%)	High: *n* (%)	
Total samples	200	100 (50.0)	100 (50.0)		100 (50.0)	100 (50.0)		100 (50.0)	100 (50.0)	
Age (years)	200	59.99 ± 8.73	62.13 ± 9.28	0.095^B^	60.58 ± 8.67	61.54 ± 9.44	0.455^B^	60.19 ± 8.57	61.93 ± 9.47	0.175^B^
Gender										
Female	74	32 (43.2)	42 (56.8)	0.094^A^	33 (44.6)	41 (55.4)	0.153^A^	34 (45.9)	40 (54.1)	0.232 ^A^
Male	126	68 (54.0)	58 (46.0)		67 (53.2)	59 (46.8)		66 (52.4)	60 (47.6)	
Tumor depth										
T1/T2	58	32 (55.2)	26 (44.8)	0.218^A^	37 (63.8)	21 (36.2)	0.009^A^	31 (53.4)	27 (46.6)	0.320^A^
T3/T4	142	68 (47.9)	74 (52.1)		63 (44.1)	79 (55.6)		69 (48.6)	73 (51.4)	
Lymph node metastasis										
Negative	85	54 (63.5)	31 (36.5)	0.001^A^	55 (64.7)	30 (35.3)	< 0.001^A^	52 (61.2)	33 (38.8)	0.005^A^
Positive	115	46 (40.0)	69 (60.0)		45 (39.1)	70 (60.9)		48 (41.7)	67 (58.3)	
Histological differentiation										
Well	60	40 (66.7)	20 (33.3)	0.007^A^	39 (65.0)	21 (35.0)	0.012^A^	43 (71.7)	17 (28.3)	< 0.001^A^
Moderate	99	44 (44.4)	55 (55.6)		46 (46.5)	53 (53.5)		41 (41.4)	58 (58.6)	
Poor	41	16 (39.0)	25 (61.0)		15 (36.6)	26 (63.4)		16 (39.0)	25 (61.0)	
Largest tumor dimension (cm)	200	4.70 ± 1.64	5.01 ± 1.22	0.135^B^	4.82 ± 1.56	5.10 ± 1.60	0.217^B^	4.84 ± 1.69	5.09 ± 1.45	0.256^B^
Stage										
I/II	78	47 (60.3)	31 (39.7)	0.029^A^	47 (60.3)	31 (39.7)	0.029^A^	43 (55.1)	35 (44.9)	0.310^A^
III	122	53 (43.4)	69 (56.6)		53 (43.4)	69 (56.6)		57 (46.7)	65 (53.3)	

High in this analysis is based on a salivary exosomal sesncRNA level or RSP higher than median; the remaining individuals were classified as low

^A^χ2 test was used for comparing control and patient group

^B^Unpaired t test was used for comparing control and patient group

**Fig. 4:**
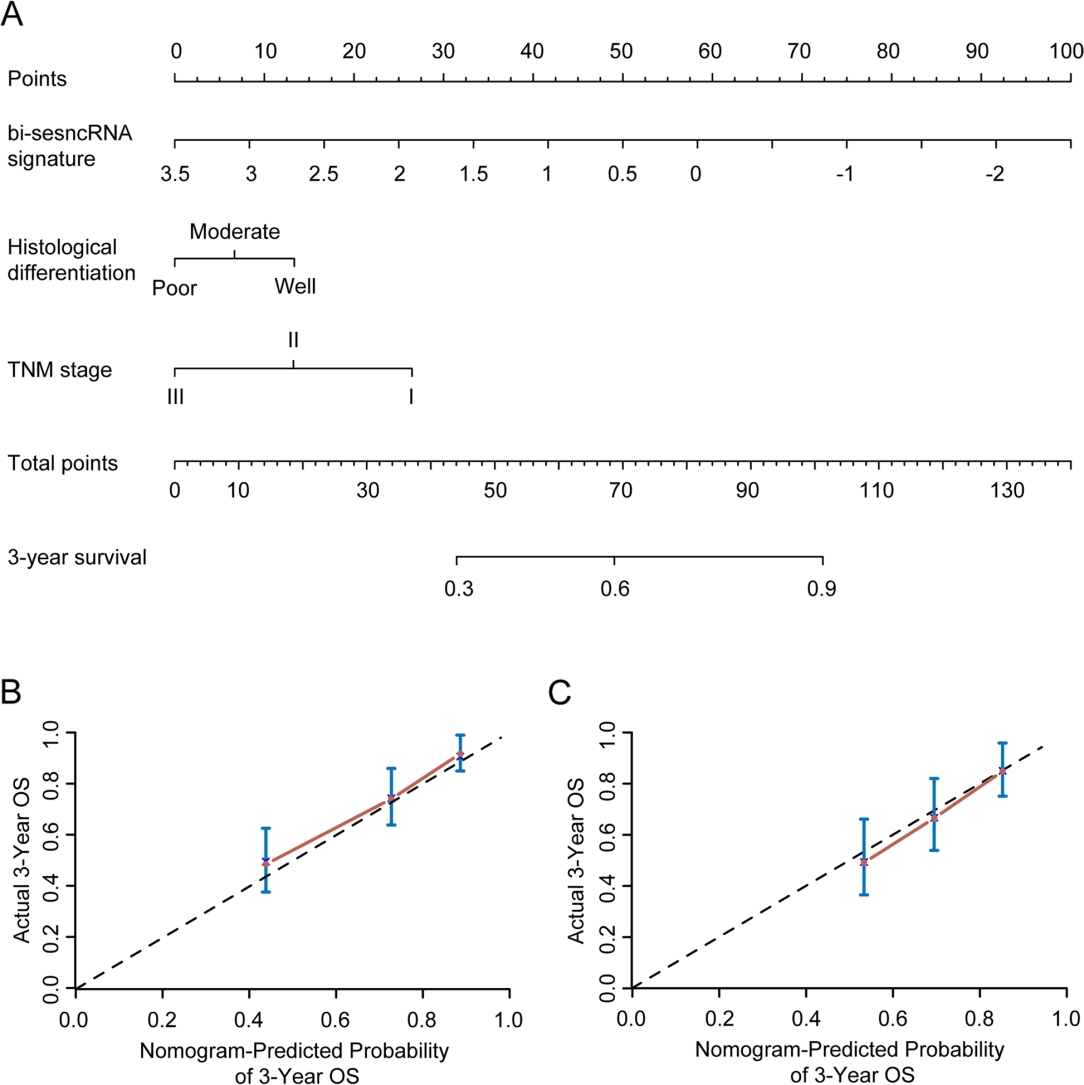
Nomogram to predict the probability of survival of ESCC patients using the bi-sesncRNAs RSP. **A** ESCC survival nomogram. The calibration curve for predicting OS at 3 years in the CHSUMC (**B**) and ATH (**C**) cohort. Nomogram-predicted probability of survival is plotted on the x-axis; actual overall survival is plotted on the y-axis

When the ESCC patients in the ATH cohort were divided into high-RSP or low-RSP groups using the above-defined cutoff value (− 0.436), Kaplan-Meier analysis revealed that compared to those with low-RSP the patients with high-RSP have shorter OS (Fig. 3D, HR = 2.06, 95%CI 1.23–3.46, *p* = 0.005) and PFS (Fig. 3H, HR = 2.05, 95%CI 1.21–3.46, *p* = 0.006), suggesting that bi-sesncRNA-derived high RSP can serve as an indicator for good prognosis of ESCC. The multivariate COX regression analysis indicates that the histological differenciation, bi-sesncRNA signature RSP and TNM stage were independent prognostic factors for OS and PFS of both CHSUMC and ATH cohorts (Table 4 and S2). To seek more precise prediction for an individual ESCC patient’s survival while controlling for TNM stage and histological differentiation, a nomogram prediction model was established based on multivariate regression analysis (Fig. 4A). The 3-year-OS were predicted well in both CHSUMC cohort (Fig. 4B, C-index = 0.718) and ATH cohort (Fig. 4C, C-index = 0.711). These findings collectively demonstrated that the bi-sesncRNA signature RSP could serve as an independent predictor for the clinical outcomes of ESCC.

**Table 4:** Univariate and multivariate Cox proportional hazards analyses of survival in ESCC patients of CHSUMC cohort

Variables	Univariate analysis		Multivariate analysis	
	HR (95% CI)	*P* value	HR (95% CI)	*P* value
Age (years)				
> 60 vs. ≤60	1.258 (0.817 to 1.936)	0.297	0.938 (0.596 to 1.475)	0.782
Gender				
Male vs. Female	0.792 (0.502 to 1.249)	0.315	0.656 (0.399 to 1.078)	0.096
Histological differentiation				
Poor vs. Well/Moderate	1.708 (1.247 to 2.339)	0.001	1.473 (1.039 to 2.088)	0.030
Largest tumor dimension (cm)				
≥ 5 vs. < 5	1.748 (1.120 to 2.728)	0.014	1.520 (0.955 to 2.421)	0.078
TNM Stage				
III vs. I/II	2.709 (1.645 to 4.463)	< 0.001	1.688 (0.985 to 2.894)	0.057
Bi-sesncRNA signature	2.062 (1.678 to 2.535)	< 0.001	1.983 (1.550 to 2.535)	< 0.001

*HR* hazard ratio, *CI* confidence interval

### Prediction of benefit of adjuvant therapy

**Fig. 5:**
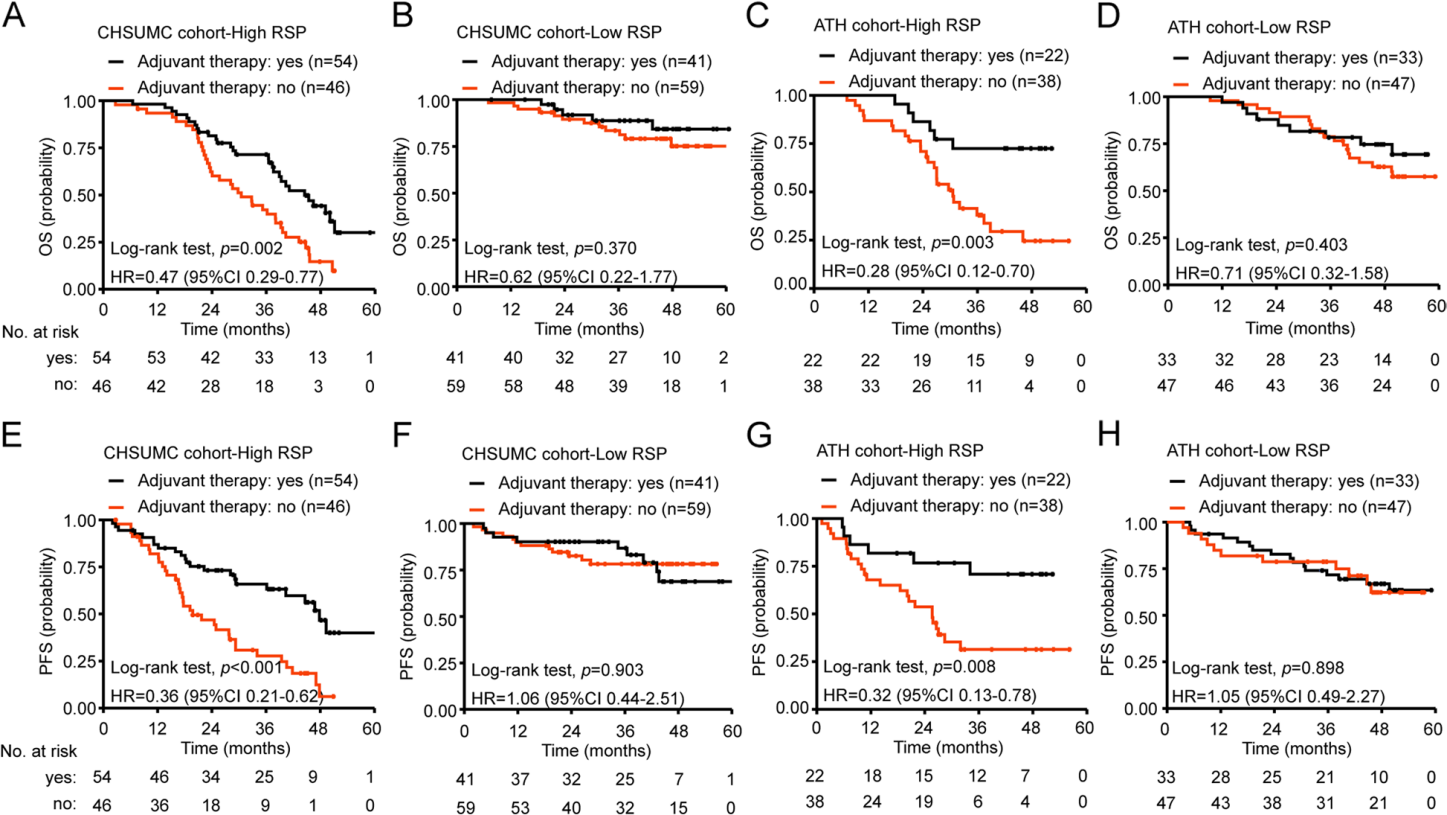
RSP for prognostication of survival and treatment prediction for postoperative therapy. **A** and **B** The benefit of postoperative therapy in patients classified as high-RSP in CHSUMC cohort. **C** and **D** The benefit of postoperative therapy in patients with low-RSP in CHSUMC cohort. **E** and **F** The benefit of postoperative therapy for patients with high-RSP in ATH cohort. **G** and **H** The benefit of postoperative therapy for patients with low-RSP in CHSUMC cohort. The *p*-values were calculated using the unadjusted log-rank test, and hazard ratios (HR) using a univariate Cox regression analysis. CI, confidence interval

We next examined the instructive role of the bi-sesncRNA signature RSP in postoperative adjuvant treatment. Using the above-established cutoff value (− 0.436) of bi-sesncRNA-derived RSP, patients were stratified into high-RSP and low-RSP groups to retrospectively analyze the effect of adjuvant therapy on ESCC clinical outcomes. In the CHSUMC cohort, 54 ESCC patients with high RSP and 41 ESCC patients with low RSP received adjuvant therapy. When the Kaplan-Meier survival analysis was stratified by the bi-sesncRNA signature RSP, we found that adjuvant therapy was associated with an improved OS (Fig. 5A, HR 0.47, 95%CI 0.29–0.77; *p* = 0.002) and PFS (Fig. 5E, HR 0.36, 95%CI 0.21–0.62; *p* < 0.001) in patients with high-RSP but not those with low-RSP value (Fig. 5B&F, OS: HR 0.62, 95%CI 0.22–1.77; *p* = 0.370; PFS: HR 1.06, 95%CI 0.44–2.51; *p* = 0.903). Similar findings were observed in the ATH cohort (High-RSP patients, OS: HR 0.28, 95%CI 0.12–0.70; *p* = 0.003; PFS: HR 0.32, 95%CI 0.13–0.78; *p* = 0.008. Low-RSP patients, OS: HR 0.71, 95%CI 0.32–1.58; *p* = 0.403; PFS: HR 1.05, 95%CI 0.49–2.27; *p* = 0.898.), in which 55 of the140 patients received adjuvant therapy (Fig. 5C-D and G-H). To avoid the influence of the bias of clinicopathological characteristics of patients with or without adjuvant therapy, χ^2^ test was performed and no significantly difference was found in these two groups (Tables S3 and S4). The results of this analysis suggested that only the ESCC patients with high bi-sesncRNA signature RSP might benefit from adjuvant therapy to improve their PFS and OS. Therefore, the bi-sesncRNA signature RSP might be a potential tool to predict which pre-operative patients might benefit from adjuvant therapy.

## Discussion

In this study, two previously uncharacterized small RNAs, tsRNA (tRNA-GlyGCC-5) and a previously unnamed small RNA (coined name: sRESE) were identified using high-throughput sequencing of small RNAs in salivary exosomes derived from ESCC patients. In a prospective clinical study, a bi-sesncRNA signature (composing of tRNA-GlyGCC-5 and sRESE) was found to serve as a non-invasive biomarker for ESCC diagnosis and prognosis as well as for prediction of adjuvant therapeutic benefits.

The bi-sesncRNA signature described here was identified by directly sequencing of exosomes derived from patients’ saliva. To trace the origin of the exosome-derived small RNAs, both tRNA-GlyGCC-5 and sRESE were validated in patients’ tissue as well as in ESCC cell lines. Furthermore, we found that ESCC cell lines secreted exosomes that contained these two small RNAs into conditioned culture media. Since the salivary exosomes of ESCC patients were enriched in these two small RNAs compared to healthy volunteers, the source or the cause of enrichment in salivary exosomes of ESCC patients was likely to be ESCC. This potential link between secretion of exosomes containing these two RNAs by ESCC cells and increased amount of them in salivary exosomes from ESCC patients suggested that their amounts in salivary exosomes might have diagnostic and/or prognostic value. Although tsRNAs in exosomes were investigated in some published studies [16, 35–37], this is the first report of salivary exosomal tsRNA as a disease biomarker.

The analysis of two prospective observational cohorts (one as training cohort and the other as validation cohort) demonstrated that a bi-sesncRNA signature RSD performed better than either small RNA alone for predicting the presence of ESCC. Interestingly, this RSD exhibited a good negative predictive value in discriminating stage I patients from healthy controls, thus a high potential for translating the bi-sesncRNA signature for future prospective ESCC screening. The bi-sesncRNA signature RSP also performed better than either small RNA alone for predicting ESCC prognosis. Although there are some diagnostic or prognostic biomarkers for ESCC already reported [38–41], the bi-sesncRNA signature exhibits high sensitivity and specificity. However, future direct head-to-head comparisons in clinical studies are required to establish superiority of the bi-sesncRNA signature for early screening and prognosis of ESCC.

Prognostic assessment is a crucial consideration in the decision to undergo adjuvant therapy. In routine clinical practice, the TNM staging of ESCC is the major prognostic determinant. However, there is still considerable variation in the clinical outcomes of ESCC patients perhaps due to heterogeneity caused by unmeasured covariates. In this study, we developed a bi-sesncRNA signature that effectively predicted the survival and therapy benefit of ESCC patients. In Cox regression models to predict OS or PFS, the HRs for the RSP and TNM stage were 1.983 and 1.688; therefore, the bi-sesncRNA signature RSP and TNM stage were both independent predictors, and the bi-sesncRNA signature is as influential as TNM stage, if not more. To our knowledge, this is the first pre-operative biomarker for predicting benefits of postoperative adjuvant therapy for ESCC. These small RNAs with their functions in promoting tumor progression are the high-risk biomarker for cancer progression after surgery. We found that high-RSP patients tend to benefit from adjuvant therapy, as elevated RSP indicating the urgent need of postoperative therapy. On the contrast, low-RSP patients did not benefit from adjuvant therapy suggesting that these patients should not be treated with these therapies after surgery to avoid the both adjuvant treatment-related side effect and financial cost. Therefore, this bi-sesncRNA signature have the potential to help clinicians develop more precise treatment plans and avoid futile or unnecessary adjuvant treatments for ESCC patients.

The salivary exosome-detection of small RNA offers several unique advantages: a) representation of the malignancy as a whole and not just the biopsy site, particular group of cancer cells or individual cancer cells compared with tissue-based assays; b) non-invasive and reproducible, and more convenient to obtain than blood; c) more layers of molecular/genetic information compared with circulating tumor DNA (ctDNA); d) less technically challenging than circulating tumor cells (CTC). Multiple proteomic, transcriptomic, and metabolomics studies have demonstrated that saliva contains biomolecules that are effective indicators of oral and systemic diseases [42, 43]. Ogawa Y et al. have investigated the small RNA profile in a healthy human salivary exosome [44], supporting that nuclear acids from salivary exosomes were stable biomarkers. Our previous study also observed that salivary exosomal chimeric RNA can serve as a non-invasive approach for molecular cancer detection, monitoring of tumor burden, and surveillance of treatment response [26]. To our knowledge, current study is the first clinical study to investigate salivary exosome-based cancer biomarkers. It is also the first clinical trial of salivary exosomal small RNAs as cancer biomarkers.

Prior to the current study, both tRNA-GlyGCC-5 and sRESE are uncharacterized especially for their functions, although tRNA-GlyGCC-5 was reported to be detected in malignant human B cells [34]. sRESE resides in chromosome 1, adjacent between *SSX2IP* and *LOC102724892*. *SSX2IP* was reported promoting metastasis and chemotherapeutic resistance in hepatocellular carcinoma and relating poor outcomes of nasopharyngeal carcinoma [45, 46], while *LOC102724892* is a lncRNA gene whose function remains unknown. This study demonstrated that both tRNA-GlyGCC-5 and sRESE promote proliferation, migration, and invasion functionally. The specific mechanisms by which tRNA-GlyGCC-5 and sRESE promote tumor progression remain incomplete and require further exploration.

One limitation of our study was that the follow-up time of many patients in our cohorts were under 5 years; therefore, our data could only support generation of the nomogram for 3-year survival. Besides, the current report is an interim analysis of the clinical trial, which may introduce uncertainty because the trial is ongoing. To avoid misleading results, more samples were enrolled for analysis and establishing the model. Upon full recruitment to the study and longer follow up time, more robust statistical analysis and generation of the nomogram for 5-year survival will be reported.

## Conclusions

In this study, we discovered a cancer-enriched bi-sesncRNA signature (i.e., tRNA-GlyGCC-5 and sRESE) in salivary exosomes, which represents a non-invasive, convenient, and reliable biomarker for human ESCC diagnosis, prognosis, and particularly, prediction of pre-operative patients who likely to benefit from adjuvant therapy. Further extensive independent prospective randomized studies are needed to validate the clinical application of this bi-sesncRNA signature.

## Supplementary Information

**Additional file 1.**

## Data Availability

All data obtained and/or analyzed in this study were available from the corresponding authors in a reasonable request.

## Abbreviations

tsRNAs: tRNA-derived small RNAs
ESCC: Esophageal squamous cell carcinoma
OS: Overall survival
PFS: Progression-free survival
sRESE: Small RNA identified in Exosome from Saliva of ESCC patients
ctDNA: Circulating tumor DNA
CTCs: Circulating tumor cells
piRNA: Piwi-interacting RNA
snoRNA: Small nucleolar RNA
tRFs: tRNA-derived fragments
sncRNAs: Small non-coding RNAs
sesncRNAs: Salivary exosomal small ncRNAs
CHSUMC: Cancer Hospital of Shantou University Medical College
ATH: Anyang Tumor Hospital
UICC: Union for International Cancer Control
TNM: Tumor-Node-Metastasis
AUROC: Area under receiver operating characteristics curve
C-index: Concordance index
NCBI: National Center for Biotechnology Information
RSD: Risk Score for Diagnosis
RSP: Risk Score for Prognosis
