# Supplementary material for: A signature of saliva-derived exosomal small RNAs as predicting biomarker for esophageal carcinoma: a multicenter prospective study
Source: Mol Cancer. 2022 Jan 18;21:21. doi: 10.1186/s12943-022-01499-8 (PMC8764835; doi:10.1186/s12943-022-01499-8)
Supplement: Supplementary file 1 — Additional file 1. [file 12943_2022_1499_MOESM1_ESM.zip › Supplementary document-without track.docx]

**Supplementary materials and methods**

**Saliva collection**

Saliva was collected in the morning (between 8 AM and 10 AM) from all subjects. Subjects were instructed to refrain from eating, drinking, or using oral hygiene products for at least 1 hour prior to collection and received no stimulation of salivation. After rinsing the mouth with water, each subject spat 3-5 ml saliva into a 35-mm dish. The subjects were reminded not to cough up mucus during collecting, and the entire process was completed within 30 minutes. The saliva sample was transferred to a 1.5-ml tube and centrifuged at 3,000×g for 15 minutes at 4 ℃ to remove cells and cellular fragments. The supernatant was transferred to a new 1.5-ml tube and stored at -80 ℃.

**Isolation of salivary exosomes**

ExoQuick^TM^ exosomes precipitation solution was used for exosomes isolation according to the manufacturer’s instructions (System Biosciences, Mountain View, Calif). Briefly, ExoQuick-TCTM solution was added to saliva at a 63/250 ratio of solution to sample and mixed by inverting the tubes at 4 ℃ overnight and then collected by centrifugation at 1,500×g for 30 minutes followed by 3000×g for 5 minutes. Next, the supernatant was discarded, and the pellet was suspended in 300μL TRIZOL for RNA isolation or 20μL phosphate-buffered saline (PBS) for protein isolation.

**Protein isolation and Immunoblotting**

Exosomal pellets were lysed in RIPA buffer (Santa Cruz Biotechnology). Total protein lysates were prepared and analyzed by immunoblotting using anti-ALIX (Cat. No.2171; Cell Signaling Technology, Beverly, MA, USA), anti-CD9 (Cat. sc-9148; Santa Cruz Biotechnology, CA, USA), anti-TSG101 (Cat. Ab133586; Abcam, Cambridge, UK), anti-CD63 (Cat. sc-15363; Santa Cruz Biotechnology, CA, USA) and anti-Calnexin (Cat. Ab241154; Abcam, Cambridge, UK) as described previously.[^1^](#_ENREF_1)^,^ [^2^](#_ENREF_2)

**Transmission electron microscopy**

Following each exosome isolation, the pellet was washed in PBS and then subjected to ultracentrifugation at 120,000×g for 70 minutes to pellet the exosomes. The exosome pellet was suspended in 30μL PBS, and a 10-μL aliquot of the suspension was loaded onto formvar carbon-coated grids and incubate for 5 minutes at room temperature. Next, the exosomes were fixed in 2% paraformaldehyde for 5 minutes at room temperature and washed thrice with PBS. Excess liquid was drained by gently touching the edge of the grid with a piece of clean filter paper. Next, the grid was dipped in 2% uranyl acetate for 1 minute and embedded in a mixture of uranyl acetate (0.8%) and methylcellulose (0.13%). Excess liquid was drained off, and then the grid was allowed to air dry for several minutes before examination under a transmission electron microscope (TEM) (JEM-1400, Hitachi, Shiga, Japan).[^1^](#_ENREF_1)^,^ [^3^](#_ENREF_3)

**Nanoparticle tracking analysis**

Total particles in human salivary exosome samples were analyzed by nanoparticle tracking (A & P Instrument Co. Ltd, Guangzhou, China), using a NanoSight LM10 system (NanoSight Ltd, Amesbury, UK). Each sample was diluted in PBS and analyzed three times. Data was collected and analyzed using the nanoparticle tracking analysis (NTA) software (RRID: SCR_014239, version 2.3). All measurements were conducted at room temperature.

**Small RNA library construction, sequencing, and data analysis**

Total RNA was extracted the salivary exosomes using TRIzol reagent per the manufacturer’s protocol (ZYMO RESEARCH). The quantity of total exosomal RNA was measured using Agilent 2100 Bioanalyzer (Agilent, Santa Clara, CA, USA) with the Bio-PicoChip. Library construction and sequencing was conducted by the Beijing Novogene Institute (Tianjin, China). Briefly, small RNAs of 18-40nt in length were purified from total RNA by size fractionation using 15% PAGE and sequentially ligated to 5’ and 3’ adaptors. Products of reverse transcription-polymerase chain reaction (RT-PCR) were used for library construction using the TruSeq Small RNA Sample Prep Kit (Illumina, Inc., San Diego, CA, USA) according to the manufacturer’s protocol. PCR products were gel-purified and sequenced using Illumina HiSeq 2000 (Illumina, Inc.). Clean reads were obtained by removing low-quality reads and adaptor sequences from raw reads. Subsequently, the length distribution of the clean reads of common and specific sequences from patients and controls were summarized. To identify differentially-expressed sesncRNAs, expression levels of each sesncRNA were normalized using the following formula: Normalized expression=actual sesncRNA count/total count of clean reads x 1,000,000. sesncRNAs with fold change ≥2.0 (log_2_ ratio ≥1.0 or ≤ - 1.0) were considered as differentially expressed.

**Reverse transcription-quantitative polymerase chain reaction (RT-qPCR)**

Total RNA was extracted from exosomes and cell lysates using TRIzol reagent as per the manufacturer’s protocol (ZYMO RESEARCH), and 25fmol of *C. elegans* cel-miR-39 RNA was added to each sample as a spike-in control.[^4^](#_ENREF_4)^,^ [^5^](#_ENREF_5) Quantification of total exosomal RNA was assessed by Agilent 2100 Bioanalyzer (Agilent, Santa Clara, CA, USA) with the Bio-PicoChip. 1.1µL of each sample was denatured for 2 minutes at 70°C, followed by a 2-minute incubation at 4°C. To obtain the total RNA profile, 1µL of each denatured sample was loaded into the chip and processed following manufacturer specifications. The final sample concentration was calculated by the Bioanalyzer. 500ng of total RNA was used for reverse transcription of small RNA by All-in-One™ miRNA First-Strand cDNA Synthesis Kit (Cat. No. QP013, GeneCopoeia, Inc., Rockville, MD, USA). RT-qPCR was performed using All-in-One™ miRNA qRT-PCR Detection Kit (Cat. No.QP015, GeneCopoeia, Inc., Rockville, MD, USA) on a Bio-Rad CFX connect real-time PCR system. The Rn value (normalized reporter value) was determined by normalizing the fluorescent signal from SYBR Green with the signal of the passive reference dye for a given reaction. No-template and no-RT reactions were performed as negative controls. We performed all assays in 3 separate RTs followed by triplicate qPCR, and the results were shown as the average fold change relative to cel-miR-39. The detailed formula used for reporting individual relative expression of each sample is 2 ^(-△CT)^= 2 ^(-(CT(individual)-CT(cel-miR-39)))^. Specific primers for RT-qPCR are:

| Uniq_198663: 5’- GATTGGTGACTGCCTAGGGCTCAGG-3’ |
| --- |
| Uniq_1434251: 5’- GCATGGGTGGTTCAGTGGTAGAATTT-3’  Uniq_1643598: 5’- GCATGATCTGAGTTCCTGTTTCTTCTG-3’  Uniq_1928942: 5’- GATTGACAAGAGGGCTGATGAACACAG-3’  Uniq_2174869: 5’- CACAGTGGGCTCTCTGTGAACTGCTGGC -3’ |

**Cell culture**

All cells were maintained at 37°C with 5% CO_2_. ESCC cells were cultured in Dulbecco’s modified Eagle’s medium (Gibco, Paisley, UK) supplemented with 10% heat-inactivated, exosome-depleted fetal bovine serum (Systembio),[^6^](#_ENREF_6) 10mmol/L glutamine, 100 units/ml penicillin (Sigma, St. Louis, MO), and 100μg/ml streptomycin (Sigma). The human immortalized esophageal epithelial cells, NE1, NE2, and NE083 were cultured in a 1:1 mixture of defined serum-free keratinocyte medium (Gibco) and EpiLife medium (Cascade Biologics). HKESC-1, HKESC-2, HKESC-3, NE1, NE2, and NE083 were kindly provided by Dr. S.W. Tsao (University of Hong Kong), KYSE22, KYSE30, KYSE70, KYSE140, KYSE150, KYSE450, KYSE510, TE7, and TE12 were obtained from the cell bank of the Chinese Academy of Medical Science, and TE1 cells were provided by Dr. X.C. Xu (The University of Texas MD Anderson Cancer Center, Houston, Texas, USA). The cells were maintained as described previously.[^7-10^](#_ENREF_7) After the authentication, the cells were immediately amplified and frozen in liquid nitrogen to serve as our stock. Although the project lasts much longer, none of these cells were sub-cultured for more than six months.

**Cell transfection with small RNA inhibitors**

Cells were transfected with small RNA inhibitors or negative control (NC), chemical synthesis oligonucleotides (Gene Pharma, China) using Lipofectamine 3000 reagent (Invitrogen, USA) according to the supplier’s instructions. The sequences of inhibitors are:

tRNA-GlyGCC-5 inhibitor: 5’- AAAUUCUACCACUGAACCACCCAUGC-3’;

sRESE inhibitor: 5’- CAGAAGAAACAGGAACUCAGAUCAUGC-3’.

**Cell viability assay**

The cell viability was assessed using the Cell Counting Kit (CCK8, MedChemExpress, HY-K0301). Briefly, KYSE150 cells and TE-12 cells were seeded into 96-well plates at an initial density of 10,000 cells per 100ul. After 24, 48, 72, and 96 hours of culture, CCK-8 solution (10ul per 100ul of the medium) was added to each well and incubated for 2h. The absorbance was measured by scanning with a microplate reader (Cytation 5 Imaging Reader, BioTek Instruments) at 450nm. Six replicates for each group and the experiment were repeated at least three times.

**Cell migration assay**

Chemotactic migration of KYSE150 cells and TE-12 cells were examined using the Transwell Chamber Assay. Cells were serum-starved for 24 h and then plated at the density of 100,000 cells per well in the serum-free medium. The RPMI 1640 medium containing 10% FBS was added into the bottom chamber as a chemoattractant. After incubation of 24 h, non-migrating cells were removed from the upper chamber, and cells migrated through the membrane were fixed with 4% formaldehyde and stained with crystal violet. Photograph of stained cells was taken using a light microscope (Leica) at a 400x magnification, and cell numbers were determined using Image J.

**Matrigel invasion assay**

The Matrigel Invasion chambers with Matrigel-coated 8-μm filters (BD) were used to study the cell invasion activity. Briefly, cells were serum-starved for 24 h and then seeded in the upper compartment of Matrigel-coated inserts. Next, the medium containing 10% FBS was added to the bottom chamber. After incubation for 24 h, non-invaded cells in the upper chamber were removed. Finally, cells that invaded through the membrane to the lower surface were stained with crystal violet and counted under a microscope (Leica).

**Supplementary references**

1. Lin Y, Dong H, Deng W, et al. Evaluation of Salivary Exosomal Chimeric GOLM1-NAA35 RNA as a Potential Biomarker in Esophageal Carcinoma. ***Clin Cancer Res***. 2019;25(10):3035-45.

2. Dong H, Ma L, Gan J, et al. PTPRO represses ERBB2-driven breast oncogenesis by dephosphorylation and endosomal internalization of ERBB2. ***Oncogene***. 2017;36(3):410-22.

3. Liao J, Liu R, Yin L, et al. Expression profiling of exosomal miRNAs derived from human esophageal cancer cells by Solexa high-throughput sequencing. ***Int J Mol Sci***. 2014;15(9):15530-51.

4. Kroh EM, Parkin RK, Mitchell PS, et al. Analysis of circulating microRNA biomarkers in plasma and serum using quantitative reverse transcription-PCR (qRT-PCR). ***Methods***. 2010;50(4):298-301.

5. Arroyo JD, Chevillet JR, Kroh EM, et al. Argonaute2 complexes carry a population of circulating microRNAs independent of vesicles in human plasma. ***Proc Natl Acad Sci U S A***. 2011;108(12):5003-8.

6. Zhang L, Zhang S, Yao J, et al. Microenvironment-induced PTEN loss by exosomal microRNA primes brain metastasis outgrowth. ***Nature***. 2015;527(7576):100-4.

7. Feng Y, Ke C, Tang Q, et al. Metformin promotes autophagy and apoptosis in esophageal squamous cell carcinoma by downregulating Stat3 signaling. ***Cell Death Dis***. 2014;5:e1088.

8. Wang L, Xiong X, Yao Z, et al. Chimeric RNA ASTN2-PAPPAas aggravates tumor progression and metastasis in human esophageal cancer. ***Cancer Lett***. 2021;501:1-11.

9. Wang L, Li K, Lin X, et al. Metformin induces human esophageal carcinoma cell pyroptosis by targeting the miR-497/PELP1 axis. ***Cancer Lett***. 2019;450:22-31.

10. Wang L, Du L, Xiong X, et al. Repurposing dextromethorphan and metformin for treating nicotine-induced cancer by directly targeting CHRNA7 to inhibit JAK2/STAT3/SOX2 signaling. ***Oncogene***. 2021;40(11):1974-87.

**Supplementary tables**

**Table S1.** Performance of bi-sesncRNA signature to differentiate stage I ESCC patients from healthy subjects in CHSUMC and ATH cohorts

| Variables | Cohorts | Cancer | Test Positive (*n*) | Test Negative (*n*) | Total (*n*) | Sensitivity | Specificity | PPV | NPV |
| --- | --- | --- | --- | --- | --- | --- | --- | --- | --- |
| Bi-sesncRNA signature | CHSUMC | Absent | 7 | 113 | 120 | 84.00% | 94.17% | 75.00% | 96.58% |
|  |  | Present | 21 | 4 | 25 |  |  |  |  |
|  |  | Total | 28 | 117 | 145 |  |  |  |  |
|  | ATH | Absent | 9 | 51 | 60 | 90.00% | 85.00% | 80.00% | 92.73% |
|  |  | Present | 36 | 4 | 40 |  |  |  |  |
|  |  | Total | 45 | 55 | 100 |  |  |  |  |
| PPV: positive predictive value; NPV: negative predictive value.  The cutoff value calculated in CHSUMC cohort was applied in the ATH cohort.  Test Positive in this analysis is based on a sesncRNA level or RSD higher than cutoff value; the remaining individuals were classified as Test Negative. | | | | | | | | | |

**Table S2.** Univariate and multivariate Cox proportional hazards analyses of survival in ESCC patients of CHSUMC cohort

| Variables | Univariate analysis | |  | Multivariate analysis | |
| --- | --- | --- | --- | --- | --- |
|  | HR (95% CI) | *P* value |  | HR (95% CI) | *P* value |
| Age (years) |  |  |  |  |  |
| >60 *vs.* ≤60 | 1.258 (0.817 to 1.936) | 0.297 |  | 0.903 (0.570 to 1.431) | 0.665 |
| Gender |  |  |  |  |  |
| Male *vs.* Female | 0.792 (0.502 to 1.249) | 0.315 |  | 0.900 (0.560 to 1.446) | 0.663 |
| Histological differentiation |  |  |  |  |  |
| Poor *vs.* Well/Moderate | 1.708 (1.247 to 2.339) | 0.001 |  | 1.422 (1.004 to 2.015) | 0.048 |
| Largest tumor dimension (cm) |  |  |  |  |  |
| ≥5 *vs.* <5 | 1.748 (1.120 to 2.728) | 0.014 |  | 1.498 (0.942 to 2.382) | 0.088 |
| TNM Stage |  |  |  |  |  |
| III *vs.* I/II | 2.709 (1.645 to 4.463) | <0.001 |  | 2.449 (1.457 to 4.117) | 0.001 |
| Bi-sesncRNA signature |  |  |  |  |  |
| High *vs.* Low | 4.951 (2.899 to 8.457) | <0.001 |  | 4.478 (2.603 to 7.704) | <0.001 |
| HR, hazard ratio; CI, conﬁdence interval.  High in this analysis is based on RSP higher than median; the remaining individuals were classified as low. | | | | | |

**Table S3** Association of postoperative treatment with demographic and clinicopathological characteristics of the CHSUMC cohort.

| Variables |  | High-RSP group | |  |  |  | Low-RSP group | |  |
| --- | --- | --- | --- | --- | --- | --- | --- | --- | --- |
|  | Patients: *n* | Yes: *n* (%) | No: *n* (%) | *P* value |  | Patients: *n* | Yes: *n* (%) | No: *n* (%) | *P* value |
| Total samples | 100 | 54 (54.0) | 46 (46.0) |  |  | 100 | 41 (41.0) | 59 (59.0) |  |
| Age (years) | 100 | 62.28+10.11 | 61.52+8.76 | 0.693^B^ |  | 100 | 62.05+9.11 | 58.90+7.99 | 0.070^B^ |
| Gender |  |  |  |  |  |  |  |  |  |
| Female | 40 | 19 (47.5) | 21 (52.5) | 0.312^A^ |  | 66 | 29 (43.9) | 37 (56.7) | 0.520^A^ |
| Male | 60 | 35 (58.3) | 25 (41.7) |  |  | 34 | 12 (35.3) | 22 (64.7) |  |
| Tumor depth |  |  |  |  |  |  |  |  |  |
| T1/T2 | 27 | 16 (59.3) | 11 (40.7) | 0.652^A^ |  | 31 | 12 (38.7) | 19 (61.3) | 0.828^A^ |
| T3/T4 | 73 | 38 (52.1) | 35 (47.9) |  |  | 69 | 29 (42.0) | 40 (58.0) |  |
| Lymph node metastasis |  |  |  |  |  |  |  |  |  |
| Negative | 33 | 22 (66.7) | 11 (33.3) | 0.090^A^ |  | 52 | 24 (46.2) | 28 (53.8) | 0.313^A^ |
| Positive | 67 | 32 (47.8) | 35 (52.2) |  |  | 48 | 17 (35.4) | 31 (64.6) |  |
| Histological differentiation |  |  |  |  |  |  |  |  |  |
| Well | 17 | 13 (76.5) | 4 (23.5) | 0.066^A^ |  | 43 | 18 (41.9) | 25 (58.1) | 0.675^A^ |
| Moderate | 58 | 31 (53.4) | 27 (46.6) |  |  | 41 | 18 (43.9) | 23 (56.1) |  |
| Poor | 25 | 10 (40.0) | 15 (60.0) |  |  | 16 | 5 (31.3) | 11 (68.8) |  |
| Largest tumor dimension (cm) | 100 | 4.76+1.26 | 5.16+1.28 | 0.121^B^ |  | 100 | 4.50+1.70 | 4.95+1.51 | 0.164^B^ |
| Stage |  |  |  |  |  |  |  |  |  |
| I/II | 35 | 22 (62.9) | 13 (37.1) | 0.213^A^ |  | 43 | 20 (46.5) | 23 (53.5) | 0.412^A^ |
| III | 65 | 32 (49.2) | 33 (50.8) |  |  | 57 | 21 (36.8) | 36 (63.2) |  |
| High in this analysis is based on a salivary exosomal sesncRNA RSP higher than median; the remaining individuals were classified as low.  Aχ2 test was used for comparing control and patient group.  BUnpaired t test was used for comparing control and patient group.  Yes: received postoperative therapy. No: not received postoperative therapy. | | | | | | | | | |

**Table S4** Association of postoperative treatment with demographic and clinicopathological characteristics of the ATH cohort.

| Variables |  | High-RSP group | |  |  |  | Low-RSP group | |  |
| --- | --- | --- | --- | --- | --- | --- | --- | --- | --- |
|  | Patients: *n* | Yes: *n* (%) | No: *n* (%) | *P* value |  | Patients: *n* | Yes: *n* (%) | No: *n* (%) | *P* value |
| Total samples | 60 | 22 (36.7) | 38 (63.3) |  |  | 80 | 33 (41.3) | 47 (58.8) |  |
| Age (years) | 60 | 61.23+7.75 | 62.98+6.28 | 0.215^B^ |  | 60 | 57.65+9.72 | 60.13+9.68 | 0.425^B^ |
| Gender |  |  |  |  |  |  |  |  |  |
| Female | 17 | 6 (35.3) | 11 (64.7) | 1.000^A^ |  | 30 | 11 (36.7) | 19 (63.3) | 0.640^A^ |
| Male | 43 | 16 (37.2) | 27 (62.8) |  |  | 50 | 22 (44.0) | 28 (56.0) |  |
| Tumor depth |  |  |  |  |  |  |  |  |  |
| T1/T2 | 35 | 12 (34.3) | 23 (65.7) | 0.787^A^ |  | 45 | 19 (42.2) | 26 (57.8) | 1.000^A^ |
| T3/T4 | 25 | 10 (40.0) | 15 (60.0) |  |  | 35 | 14 (40.0) | 21 (60.0) |  |
| Lymph node metastasis |  |  |  |  |  |  |  |  |  |
| Negative | 29 | 8 (27.6) | 21 (72.4) | 0.188^A^ |  | 49 | 19 (38.8) | 30 (61.2) | 0.644^A^ |
| Positive | 31 | 14 (45.2) | 17 (54.8) |  |  | 31 | 14 (45.2) | 17 (54.8) |  |
| Histological differentiation |  |  |  |  |  |  |  |  |  |
| Well | 19 | 5 (26.3) | 14 (73.7) | 0.091^A^ |  | 25 | 8 (32.0) | 17 (68.0) | 0.420^A^ |
| Moderate | 30 | 15 (50.0) | 15 (50.0) |  |  | 48 | 21 (43.8) | 27 (56.3) |  |
| Poor | 11 | 2 (18.2) | 9 (81.8) |  |  | 7 | 4 (57.1) | 3 (42.9) |  |
| Largest tumor dimension (cm) | 60 | 4.05+1.33 | 4.98+1.65 | 0.164^B^ |  | 80 | 5.01+1.48 | 5.34+1.95 | 0.223^B^ |
| Stage |  |  |  |  |  |  |  |  |  |
| I/II | 28 | 10 (35.7) | 18 (64.3) | 1.000^A^ |  | 49 | 23 (46.9) | 26 (53.1) | 0.246^A^ |
| III | 32 | 12 (37.5) | 20 (62.5) |  |  | 31 | 10 (32.3) | 21 (67.7) |  |
| High in this analysis is based on a salivary exosomal sesncRNA RSP higher than median; the remaining individuals were classified as low.  Aχ2 test was used for comparing control and patient group.  BUnpaired t test was used for comparing control and patient group.  Yes: received postoperative therapy. No: not received postoperative therapy. | | | | | | | | | |

**Supplementary figure legends**

**Figure S1.** Isolation of salivary exosomes in ESCC patients and healthy subjects. A, transmission electron micrograph of exosomes purified from human saliva. Scale bar, 200nm. B, immunoblotting of exosomal membrane markers in exosomes purified from human saliva. C, exosomes concentration and size distribution by NanoSight analysis of human saliva.

**Figure S2.** The levels of sesncRNAs in exosomes (A) from the cell-conditioned culture media compared to the lysates (B) of ESCC cell lines. A, RT-qPCR analysis of tRNA-GlyGCC-5 (left) and sesncRNAs (right) level in exosomes derived from a panel of ESCC cell lines (filled bars) and immortalized normal esophageal epithelial cell lines (open bar). B, RT-qPCR analysis of tRNA-GlyGCC-5 (left) and sRESE (right) level in cell lysates from the same panel of cells in A.

**Figure S3.** Confirmation of sesncRNAs RT-PCR product by Sanger sequencing. Salivary exosomes, tumor tissue of ESCC patients, and ESCC cell lines were used to extract RNA. (A) Gel-electrophoresis of RT-PCR products of two sesncRNAs. (B) Sanger sequencing of the PCR products.

**Figure S4.** sesncRNAs promote ESCC cell proliferation, migration and invasion. A, TE12 cell migration and invasion after transfecting with antisense RNA or scrambled controls. Cell number in the field (400×) was shown in the bottom panel. B, KYSE150 or TE12 cells were transfected with antisense RNA or scrambled controls. Cell proliferation was estimated by MTT assay. All the experiments were performed in triplicate. SEM (**p* < 0.05, ***p* < 0.01, ****p* < 0.001, *****p* < 0.0001 by unpaired t-test).
