# Supplementary figures and images for: A signature of saliva-derived exosomal small RNAs as predicting biomarker for esophageal carcinoma: a multicenter prospective study
Source: Mol Cancer. 2022 Jan 18;21:21. doi: 10.1186/s12943-022-01499-8 (PMC8764835; doi:10.1186/s12943-022-01499-8)

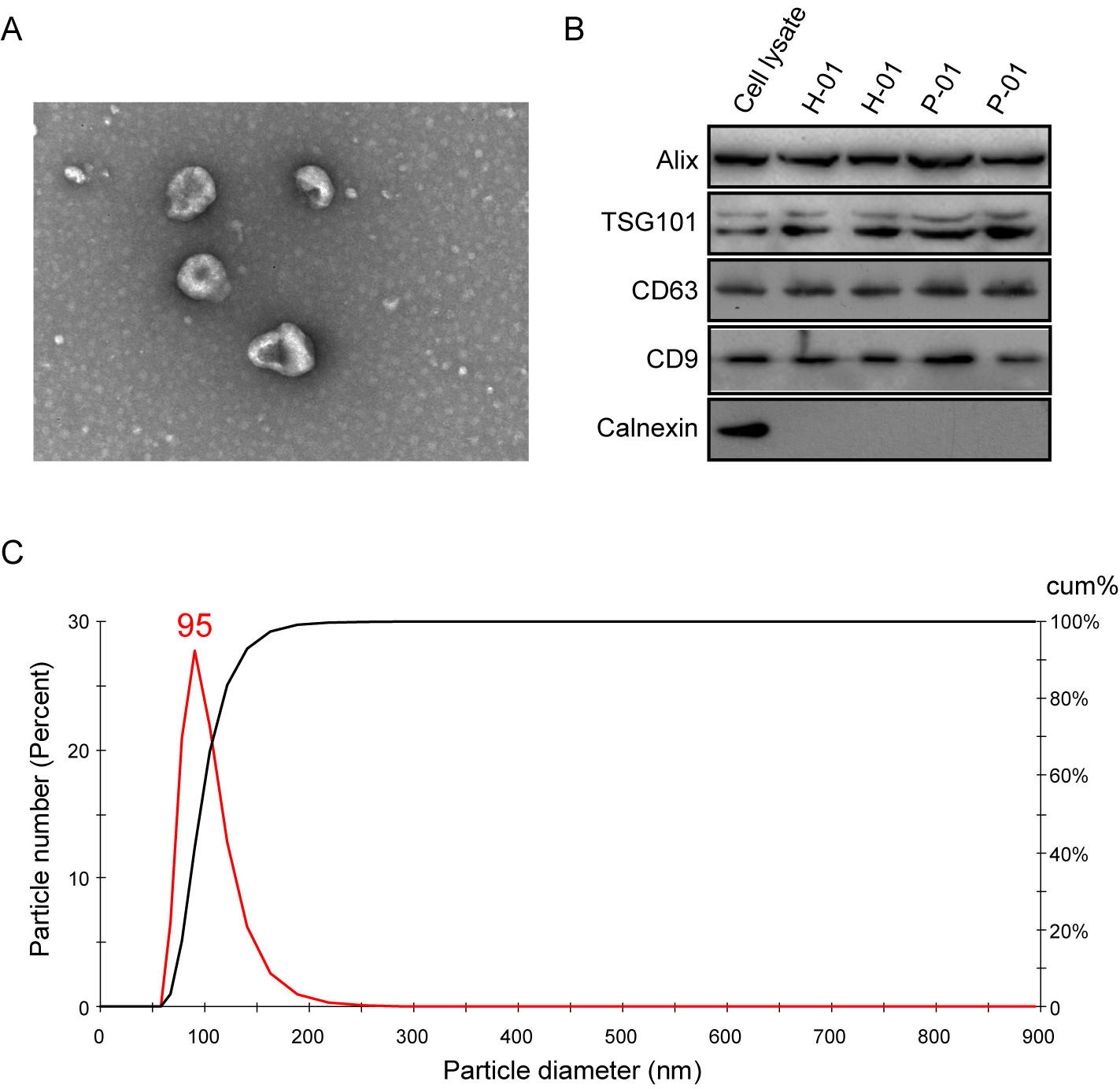

Supplement: Supplementary file 1 — Additional file 1. [file 12943_2022_1499_MOESM1_ESM.zip › Figure S1.tif]

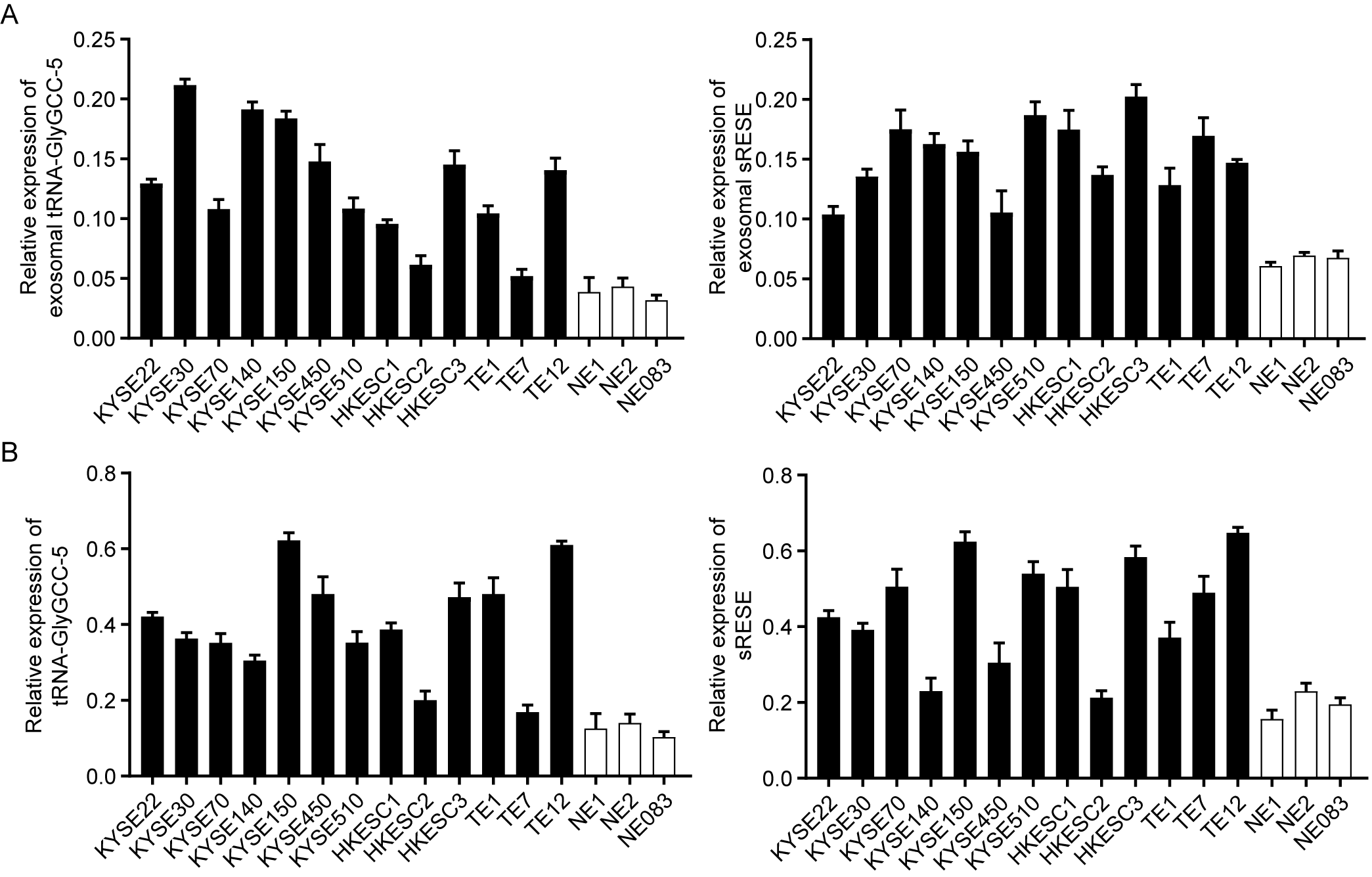

Supplement: Supplementary file 1 — Additional file 1. [file 12943_2022_1499_MOESM1_ESM.zip › Figure S2.tif]

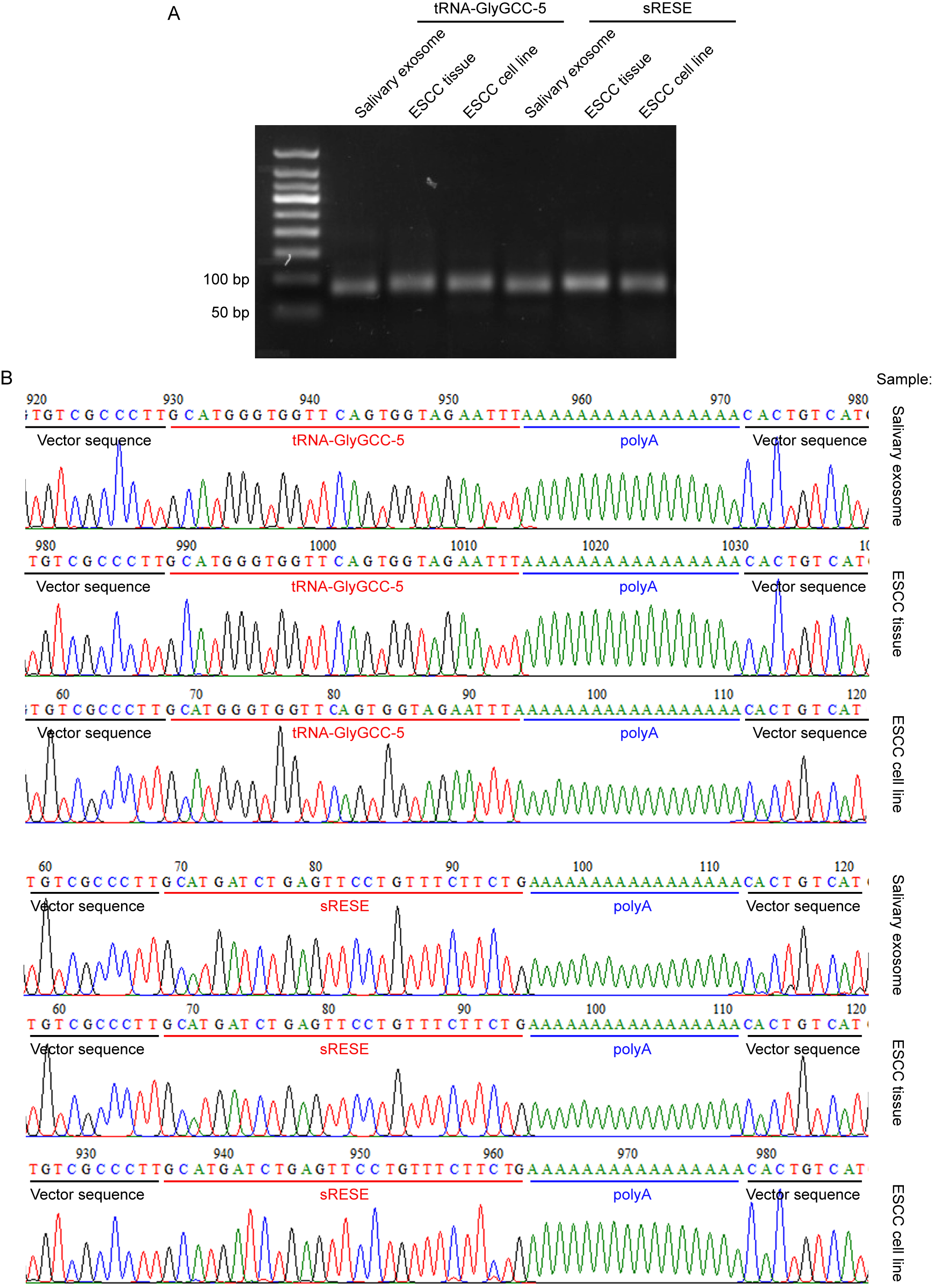

Supplement: Supplementary file 1 — Additional file 1. [file 12943_2022_1499_MOESM1_ESM.zip › Figure S3.tif]

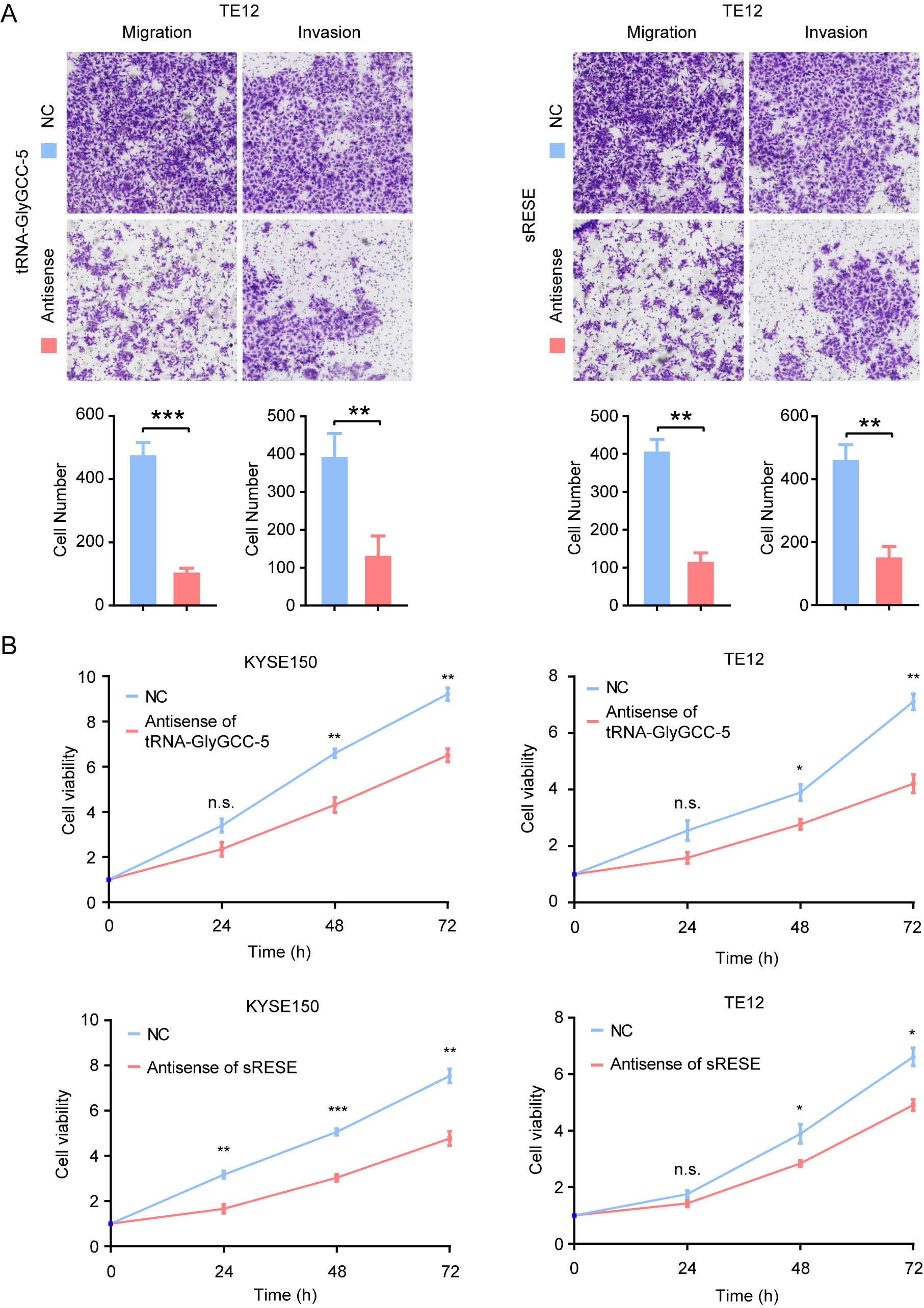

Supplement: Supplementary file 1 — Additional file 1. [file 12943_2022_1499_MOESM1_ESM.zip › Figure S4.tif]
